# Malaria in Pregnancy: What Can the Social Sciences Contribute?

**DOI:** 10.1371/journal.pmed.0040092

**Published:** 2007-04-10

**Authors:** Joan Muela Ribera, Susanna Hausmann-Muela, Umberto D'Alessandro, Koen Peeters Grietens

## Abstract

Building on existing knowledge from social science work on malaria, the authors propose two models for studying social science aspects of malaria in pregnancy.

## Introduction

Social science literature on malaria and its control is abundant. However, nearly all the publications focus on children under the age of five. Even in gender-oriented literature, women are depicted as “mothers and caretakers of children” rather than as women suffering from malaria. The specific topic of malaria in pregnancy has received little attention in social science literature, with only some 20 articles explicitly integrating social science aspects [1–3].

Currently, the recommended intervention strategies for preventing malaria during pregnancy are intermittent preventive treatment (IPT) with sulfadoxine-pyrimethamine and insecticide-treated bed nets [4]. However, in many African countries, the coverage of such interventions varies from modest to extremely low [5,6]. Although reports repeatedly mention the need to focus on behavioural aspects to better reach pregnant women, little has been done to actually promote such studies. Furthermore, though the intervention studies mention “vulnerable groups”, “utilisation of health-care services”, “delay”, or “beliefs” as important factors for effective prevention and treatment—all wordings which should immediately call social scientists onstage—behavioural and other social science research going beyond simplistic knowledge, attitudes, and practices studies is largely absent. Fortunately, interest in social science studies on malaria in pregnancy is slowly awakening.

Building on already existing knowledge from social science work on malaria, we propose two models for studying social science aspects of malaria in pregnancy. In the extended version of this paper (see Supplementary Information), we complement the two models with a literature review about recognition of malaria and anaemia in pregnancy, utilisation of antenatal clinics, acceptance of chemoprophylaxis and IPT, and adolescent pregnancy, and we provide further theoretical references about basic models used in health-seeking behaviour.

## The Malaria in Pregnancy Models: Factors Influencing Malaria in Pregnancy

**Figure 1:**
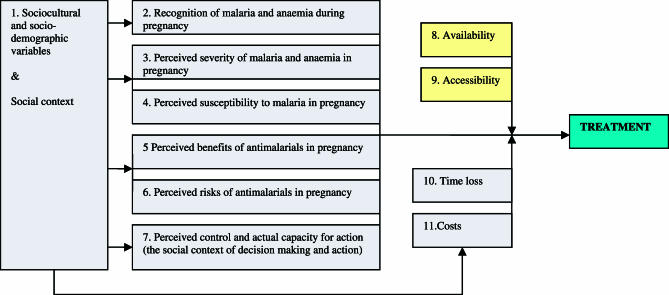
The Malaria in Pregnancy Treatment Model: Relevant Factors for Treatment-Seeking Behaviour for Malaria in Pregnant Women

**Figure 2:**
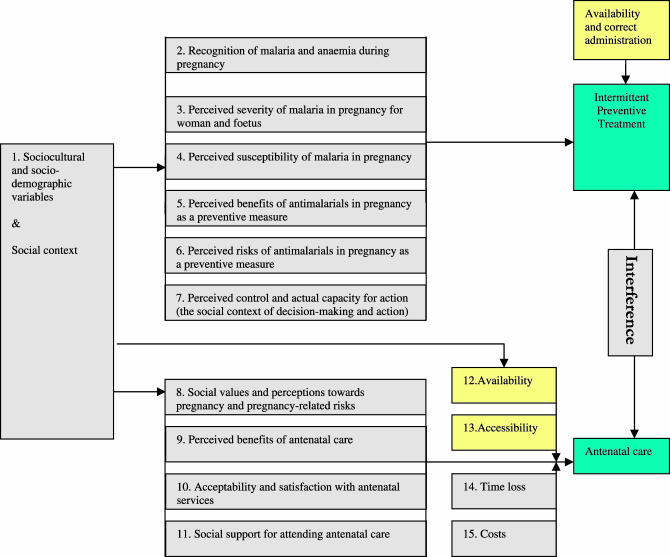
The Malaria in Pregnancy Prevention Model: Relevant Factors for the Utilization of ANCs and Acceptance of IPT

We propose two models for studying the social science aspects of malaria in pregnancy: the “Malaria in Pregnancy Treatment Model” and the “Malaria in Pregnancy Prevention Model” (see Figures 1 and 2). These models are a conglomerate of different psycho-social and socio-behavioural models, based on our own field research experience and on a literature review. We believe that these adapted models comprehensively and holistically describe the most relevant factors involved in malaria and pregnancy.

For reasons of comprehensiveness, we have limited our models to (A) treatment and (B) prevention with IPT through antenatal clinics (ANCs). The two should be considered basic, potentially generic models, adaptable to variations beyond malaria and pregnancy.

## The Treatment Model

### 1. Socio-cultural and demographic variables and social context.

The socio-cultural and demographic variables help to distinguish different groups, according to age/age group, number of pregnancies, socio-economic status, marital status, religion and magico-religious beliefs, and ethnicity, and can include other factors which may differentiate groups of people according to relevant criteria. The list is not complete, but rather gives the classical variables that serve as a basis for designating specific social categories that can be compared and possibly individually targeted. Depending on the specific setting, other socio-cultural and demographic variables might be relevant.

### 2. Recognition of malaria and anaemia during pregnancy.

While community knowledge about the malaria–mosquito link can be considered fundamental in determining the use of insecticide-treated bed nets, prompt and effective treatment depends on illness recognition. However, malaria-related symptoms can be easily confused with pregnancy-related symptoms. No publication was found that explores the way pregnant women distinguish malaria signs from general malaise and other common symptoms (e.g., nausea, vomiting, weakness, etc.) during pregnancy. Furthermore, it is necessary to determine whether pregnant women's susceptibility to anaemia is commonly known, and if people realize the link between anaemia during pregnancy and malaria.

### 3. Perceived severity.

Studies have shown that malaria is often not perceived as severe, but rather as a mild, self-limiting illness which does not require immediate treatment [7,8]. Studies should focus on (1) the perceived severity of malaria for the mother, with emphasis on knowledge regarding anaemia and maternal mortality risk; (2) the perceived severity of malaria for the foetus, and the recognition of the risk for miscarriage; and (3) the perceived severity of the illness for the newborn, including the association of malaria with low birth weight and increased vulnerability to other illnesses.

### 4. Perceived susceptibility.

With regard to malaria, perceived susceptibility is related to two factors: (1) the perceived propensity to develop clinical malaria due to idiosyncratic features of the person (pregnant women, children, immune-compromised persons); and (2) the perceived level of exposure. For instance, in places where malaria is associated with mosquitoes and rainfall [9,10], perceived susceptibility of contracting malaria seems to be strongly related to mosquito density and the rainy season [11]. These factors necessarily lead to two research questions regarding perceived susceptibility to malaria during pregnancy: (1) whether pregnant women are considered particularly susceptible to malaria and (2) when are they perceived to be more susceptible (i.e., related to seasonality or to risk activities).

### 5. Perceived benefits.

Perceived benefits need to be studied in relation to (1) the perceived efficacy of a product or an intervention (determined not only by the empirical experience, but also by the persuasiveness of the message); and (2) the perceived costs/benefits, understood not only in economic and health-related terms, but also socially and psychologically. In this sense, factors such as the evaluation of the distance to treatment facilities, the waiting time, or the behaviour of health staff can play an important role.

Similar to perceived severity, perceived benefits can be identified (1) for the mother; (2) for the foetus; and (3) for the newborn. It is important to know whether socially the mother or the foetus is prioritized since the benefits for one might imply risks for the other.

Perceived benefits should always be studied taking into consideration their counterpart, the perceived risks.

### 6. Perceived risks.

Perceived risks of treatment are among the central factors for understanding treatment acceptance and use. Just as with perceived severity and perceived benefits, perceived risks refer to mother, foetus, and newborn. Perceived risks are related to (1) perceived iatrogenic effects of treatment; (2) perceived side effects of antimalarials; and (3) perceived risks of under- and over-dosage of antimalarials. An example of a perceived risk is the fear of adverse drug reactions if the pregnant woman is possessed by a spirit that rejects Western pharmaceuticals.

Additionally, the risk from the biomedical perspective might not be considered a real threat by the affected individual, or the perceived risk may be offset by perceived complementary benefits. For example, in Burkina Faso preliminary qualitative information suggests that women prefer low birth weight for their babies since they are considered easier to deliver and “small babies” have the perceived benefit of reducing the risk of episiotomy. Women state they prefer “the baby to grow after giving birth instead of before” (Peeters Grietens, personal communication).

### 7. Perceived control and decision-making.

Perceived control and decision-making should not be understood as an individual factor but rather as a space where different factors come into play. A tension exists, and must be taken into consideration, between (1) the perceived control over action, determined by perceived and real access to the necessary resources for successful action (information, assets, abilities, social networks, opportunities, etc.), and the value attributed to these resources [12] and (2) the perceived obstacles and structural limitations (social, institutional, economic) to the planned action. In order to elaborate on this, we must focus on the people who advise and decide on the conduct of the pregnant woman—what Janzen terms the “Therapy Management Group” [13]. For instance, when women as the main caretakers recognise illness but decision-making and control over resources (e.g., money for covering transport costs) are in the hands of their husbands, who possibly have other priorities, perceived necessity and action do not correspond, presenting a similar situation to that described for childhood malaria [14].

It is important to study how decisions for malaria treatment seeking in pregnant women are made, to understand the intra-household hierarchies and the criteria implied in decision-making. In this way, participation may be strongly influenced by others than those directly targeted. It is also important to take into account the social pressure for complying with the treatment (or preventive measures), determined by gender ideologies which can result in stigmatisation (“the bad mother”), and socio-moral perceptions about “appropriateness” and “responsibility” [15,16].

### 8–10. Availability, accessibility, and time loss.

Availability, accessibility, and time loss are important factors accounting for therapeutic delay. Availability implies, among other things, that the health facilities are equipped with drugs and are recognised as competent for diagnosis and treatment. Concerning access to treatment, it should be noted that rural women may need to work and live on fields situated far away from the health centres and, during the rainy seasons, roads may be inaccessible, hindering access to health centres. The perception of time lost travelling to and from the health centre or waiting at the health centre clearly influences treatment seeking in relation to the work situation of the women, with regard to child care and intra-domestic labour substitution. Perception of time lost pertains to perceived and real loss of productivity during treatment seeking and, at the same time, is linked to the perceived impact and severity of the illness.

### 11. Direct and indirect costs.

The few studies that contemplate costs only consider the medical costs involved in treatment, e.g., [17]. However, other non-medical direct and indirect costs are essential to understanding the total costs of a malaria episode. Such non-medical direct costs consist of transportation costs, costs for the accompanying relatives, food for the patient and visitors, payment for labour substitution, etc. Indirect costs refer to productivity loss of the patient and caretaker(s), encompassing time lost travelling to a health centre and work lost while hospitalised, both for patients and their caretakers [18,19].

## Prevention with IPT

Prevention with IPT represents an intervention that encompasses two levels: the administration of IPT and the structure of antenatal care (Figure 2). For the IPT part, the above mentioned factors 1–7 practically remain unaltered and are not repeated here. There are only a few alterations with regard to antimalarials used as a preventive rather than treatment measure.

Factors 12–15 in Figure 2—availability, accessibility, time loss, and cost factors—are similar to those mentioned in the treatment model (Figure 1), with the difference that they directly relate to ANCs rather than to malaria treatment and perceived adequacy of curative care. It is worth emphasising that even when ANCs are free of charge, direct non-medical and indirect costs, especially those for transport, are still considerable and might hinder access.

In the following section, we describe the factors relevant at the ANC level, the factors 8–12 in Figure 2.

### 8. Social values, perception, and attitudes towards pregnancy and pregnancy-related risks.

ANC facilities are public spaces, where women unavoidably reveal their pregnancy to other community members. Fear of gossip and humiliation due to socially unacceptable and shameful pregnancies, e.g., adolescent or out-of-wedlock pregnancies, can be a reason for delaying or even avoiding prenatal care altogether (Peeters Grietens, unpublished report). Another important reason for avoiding public exposure of their pregnancy during the first months is the fear of sorcery-related harm to mother or foetus [20].

A further key factor is the perception of pregnancy and pregnancy-related risks. These perceptions are based on (1) cultural models of the physiology of pregnancy and foetal growth, including the symptoms of “normal” pregnancy and (2) social values concerning “appropriate” behaviour, e.g., if it is socially acceptable to publicly show suffering or fatigue. Perceptions of risks together with perceived benefits constitute the perceived need to attend ANCs.

### 9. Perceived benefits of antenatal care.

It is important to know women's perception of the different services offered at ANCs—control of foetal development; control of infectious diseases such as AIDS and other STDs; preventive measures such as administration of iodine, iron, and folic acid complements; tetanus immunisation; breast-feeding counselling and birth preparation—since some of these services might be valued more than others. Some services might encourage while others might discourage ANC attendance.

The perceived benefits of ANCs should be studied, taking into consideration perceived obstacles for attendance. Obstacles, e.g., work load, lack of transport, or limited amount of money, may be more of a deterrent for routine visits than for visits under circumstances of perceived risk.

### 10. Acceptability of and satisfaction with antenatal services.

Acceptability, particularly satisfaction with ANCs, is usually related to (1) the perceived behaviour of health personnel and (2) the perceived quality of services. Common complaints on health personnel misbehaviour include claims of medical neglect, patient humiliation, and rudeness at the hands of medical staff [21]. Low acceptability can also be related to ethnic or class differences between health staff and patients and can result in misunderstandings and mutual mistrust. Communication barriers due to different vernacular languages or to women's fear—particularly among Muslims—of being attended by male nurses can further discourage ANC attendance [22].

### 11. Social support for attending ANCs.

With regard to access to ANCs, social network support has two important dimensions: (1) social support: when giving advice to the pregnant woman; participating in pregnancy rituals where they exist; accompanying the woman to antenatal services etc.; and, (2) economic support: contributing to ANC costs and other direct and indirect costs. It is important to know who participates in which dimension, who decides to attend ANCs and how these decisions are reached. Since pregnancy is a family matter, both dimensions must be understood in the framework of social organisation of procreation, and of gender and kinship ideologies. Lack of social support can be due to various factors, e.g., impoverished households, absent “relevant others”, or poor social pressure for the husband to pay [23].

## Interference

The great difference of the prevention model when compared to the treatment model is the overlapping of ANC utilisation and IPT acceptance and the probability of interaction between both sets of variables.

Logistically, the combination of control methods like the integration of IPT into ANC structures is highly advantageous. However, the effects of one on the other are often underestimated. From the health provider side, the workload and required knowledge might mean a considerable additional burden for the health personnel. From the pregnant women's side, interferences are likely but largely unknown (in Figure 2 depicted as “interference”). Indications that such interference and community response exist comes from a study carried out in Malawi where community distribution of IPT through peer educators from the adolescent literacy programme led to a decrease in formal ANC attendance and hospital delivery (Bernard Brabin, personal communication). Similarly, IPT delivered through ANCs might encourage or discourage further attendance and influence women's perception of contracting malaria in pregnancy.

## Conclusions

We have proposed two models which encompass the different social factors that influence health-seeking behaviour for malaria in pregnant women and demonstrate how they are related to each other. Together with quantitative analysis, which evaluates the relative occurrence of the different factors and ranks them according to their frequency, qualitative analysis is incorporated to contextualise the factors, put them into a dynamic relation, and assess their relative weight and importance within the general social structure. We have developed these models for malaria in pregnancy because it is in this field that social sciences are particularly neglected. Research on and implementation of malaria control intervention for pregnant women have predominantly ignored community responses or, when considered, they have centred on single, isolated factors usually with the aim of designing “culturally sensitive” information, education, and communication messages. Reality, however, shows that the implementation of “simple” tools, like IPT delivery to pregnant women, is not so simple because community reactions are not taken into account.

With this article, we hope to create awareness among researchers of such complex interactions and the need of involving social sciences even for apparently “straightforward” interventions. Studying these interactions may help to improve the delivery of adequate interventions and thus contribute to reaching the Abuja Malaria Summit target of at least 60% of pregnant women adequately protected against malaria infection and its consequences. These models have to be taken as a first contribution to concisely cover social science aspects. We hope that they help to inspire future works in the malaria social science literature, particularly in the under-researched field of malaria and pregnancy.

## Supplementary Information

Alternative Language Abstract S1 Translation of an abstract of this article into Spanish by JMR (6 KB PDF).

Text S2 Longer version of this article (128 KB DOC).

## Abbreviations

ANC: antenatal clinic
IPT: intermittent preventive treatment

## References

[pmed-0040092-b001] McCombie SC (1996). Treatment seeking for malaria: A review of recent research. Soc Sci Med.

[pmed-0040092-b002] Williams HA, Jones C (2004). A critical review of behavioural issues related to malaria control in sub-Saharan Africa: What contributions have social scientists made?. Soc Sci Med.

[pmed-0040092-b003] Heggenhougen HK, Hackenthal V, Vivek P (2003). The behavioural and social aspects of malaria and its control. http://www.who.int/tdr/publications/publications/seb-malaria.htm.

[pmed-0040092-b004] World Health Organization (2004). A strategic framework for malaria prevention and control during pregnancy in the African region. http://www.cdc.gov/malaria/pdf/strategic_framework_mip_04.pdf.

[pmed-0040092-b005] Holtz TH, Kachur SP, Roberts JM, Marum LH, Mkandala C (2004). Use of antenatal care services and intermittent preventive treatment for malaria among pregnant women in Blantyre District, Malawi. Trop Med Int Health.

[pmed-0040092-b006] Guyatt HL, Noor AM, Ochola SA, Snow RW (2004). Use of intermittent presumptive treatment and insecticide treated bed nets by pregnant women in four Kenyan districts. Trop Med Int Health.

[pmed-0040092-b007] Mwenesi H, Harpham T, Snow RW (1995). Child malaria treatment practices among mothers in Kenya. Soc Sci Med.

[pmed-0040092-b008] Winch PJ, Makemba AM, Kamazima SR, Lurie M, Lwihula GK (1996). Local terminology for febrile illnesses in Bagamoyo District, Tanzania, and its impact on the design of a community based malaria control programme. Soc Sci Med.

[pmed-0040092-b009] Minja H (2001). Introducing insecticide treated mosquito nets in the Kilombero Valley (Tanzania): Social and cultural dimensions [PhD thesis].

[pmed-0040092-b010] Winch PJ, Makemba AM, Kamazima SR, Lurie M, Lwihula GK (1994). Seasonal variation in the perceived risk of malaria: Implications for the promotion of insecticide-treated nets. Soc Sci Med.

[pmed-0040092-b011] Sauerborn R, Nougtara A, Hien M, Diesfeld H J (1996). Seasonal variations of household costs of illness in Burkina Faso. Soc Sci Med.

[pmed-0040092-b012] Conner M, Sparks P, Conner M, Norman P (1995). The theory of planned behaviour and health behaviours. Predicting health behaviour.

[pmed-0040092-b013] Janzen JM (1978). The quest for therapy in lower Zaire.

[pmed-0040092-b014] Mwenesi H (1993). Mother's definition and treatment of childhood malaria on the Kenyan coast [PhD thesis].

[pmed-0040092-b015] De Martin S, Seidlein L, Deen JL, Pinder M, Walraven G, Greenwood B (2001). Community perceptions of a mass administration of an antimalarial drug combination in The Gambia. Trop Med & Int Health.

[pmed-0040092-b016] Ager A (1992). Perception of risk for malaria and schistosomiasis in rural Malawi. Trop Med Parasitol.

[pmed-0040092-b017] Manzi F, Armstrong Schellenberg J, Adam T, Mshinda H, Victoria CG (2005). Out-of-pocket payments for under-five health care in rural southern Tanzania. Health Policy Plan.

[pmed-0040092-b018] Russell S (2004). The economic burden of illness for households in developing countries: A review of studies focusing on malaria, tuberculosis and human immunodeficiency virus/acquired immunodeficiency syndrome. Am J Trop Med and Hyg.

[pmed-0040092-b019] McIntyre D, Thiede M, Dahlgren G, Whitehead M (2006). What are the economic consequences for households of illness and of paying for health care in low- and middle-income country contexts?. Soc Sci Med.

[pmed-0040092-b020] Mainbolwa M, Yamba B, Divan V, Ransjö-Arvidson AB (2003). Cultural childbirth practices and beliefs in Zambia. Issues Innov Nurs Pract.

[pmed-0040092-b021] Pires Lucas d'Oliveira AF, Grilo Diniz S, Blima Schraiber LB (2002). Violence against women in health-care institutions: An emerging problem. Lancet.

[pmed-0040092-b022] Zanconato G, Msolomba R, Guarenti L, Franchi M (2006). Antenatal care in developing countries: The need for a tailored model. Semin Fetal Neonatal Med.

[pmed-0040092-b023] Telfer ML, Rowley JT, Walraven GEL (2002). Experiences of mothers with antenatal, delivery and postpartum care in rural Gambia. Afr J Reprod Health.

