# Supplementary material for: Malaria in Pregnancy: What Can the Social Sciences Contribute?
Source: PLoS Med. 2007 Apr 10;4(4):e92. doi: 10.1371/journal.pmed.0040092 (PMC1851625; doi:10.1371/journal.pmed.0040092)
Supplement: Supplementary file 1 [file pmed.0040092.sd001.doc]

**Versión en español**

**RESUMEN**

**“PALUDISMO EN EL EMBARAZO: ¿QUÉ PUEDEN APORTAR LAS CIENCIAS SOCIALES?”**

Aunque la literatura sobre los aspectos sociales del paludismo y su control abunda, casi todas las publicaciones se centran en los niños menores de cinco años. En el campo específico del paludismo durante el embarazo sólo unos veinte artículos incluyen explícitamente aspectos socioculturales. Incluso en la literatura de género las mujeres se perfilan más como ‘madres y cuidadoras’ de niños enfermos que como personas que sufren la enfermedad.

Las estrategias recomendadas en la actualidad para prevenir el paludismo durante el embarazo son el Tratamiento Intermitente Preventivo (TIP) con Sulfadoxina-Pirimetamina (SP) y las telas Mosquiteras Impregnadas con Insecticida (MII). De todos modos, la cobertura de estas intervenciones en el África Subsahariana varía de moderada a muy baja. Aunque los informes mencionan reiteradamente la necesidad de tener en cuenta los aspectos conductuales para mejorar el acceso de las mujeres embarazadas a los pogramas de salud, poco se ha hecho para promocionar realmente tales estudios. Además, a pesar de que los estudios mencionan ‘grupos vulnerables’, ‘uso de los servicios de salud’, ‘retraso’ o ‘creencias’ como factores importantes a tener en cuenta cuando se estudia la prevención y el tratamiento del paludismo, conceptos todos ellos que deberían hacer pensar en la necesidad de establecer proyectos con un sólido componente en ciencias sociales, carecemos de estudios que vayan más allá de la perspectiva simplista que a menudo aporta la metodología de los CAP (de ‘conocimiento’, ‘actitudes’ y ‘prácticas’). Afortunadamente nos encontramos ante un interés creciente - aunque lento - por las ciencias sociales en el área del paludismo durante el embarazo.

A partir de los trabajos realizados en paludismo y embarazo, nuestra propia investigación y los modelos socioconductuales de procesos asistenciales y acceso a los centros de salud, proponemos dos modelos para estudiar el acceso de las mujeres embarazadas (1) al tratamiento con fármacos antipalúdicos, y (2) a la quimioprofilaxis (específicamente el TIP).

En el primer modelo presentamos y discutimos una serie de factores clave, como el problema del reconocimiento del paludismo y la anemia durante el embarazo, la gravedad percibida de la enfermedad, la susceptibilidad percibida a contraer el paludismo, los beneficios y riesgos percibidos del tratamiento, y otros factores determinantes del acceso, como los costes, la distancia, el tiempo de espera y los procesos de toma de decisiones.

En cuanto que el TIP es una estrategia que tiene lugar en los centros de salud antenatal, en el segundo modelo contemplamos aquellos factores que inciden en el acceso a estos servicios. Incluimos los valores sociales relacionados con el embarazo, la aceptabilidad y satisfacción con los servicios antenatales y el apoyo social como un factor clave de acceso. Enfatizamos la necesidad de estudiar los posibles efectos del TIP sobre el acceso a los cuidados antenatales y otras interferencias que puedan resultar de incluir el TIP en los servicios de atención antenatal.

En la versión completa del artículo (formato PDF) presentamos una revisión bibliográfica sobre reconocimiento del paludismo durante el embarazo, uso de las clínicas de atención antenatal, aceptación de la quimioprofilaxis y TIP, y la problemática de las adolescentes embarazadas, en cuánto que, en términos de acceso a los cuidados antenatales, constituyen un grupo social especialmente vulnerable. En el artículo completo incluimos además algunas referencias teóricas a los modelos socioconductuales que han nutrido tanto el modelo terapéutico como el de prevención con TIP en los servicios antenatales.
