# Supplementary material for: Malaria in Pregnancy: What Can the Social Sciences Contribute?
Source: PLoS Med. 2007 Apr 10;4(4):e92. doi: 10.1371/journal.pmed.0040092 (PMC1851625; doi:10.1371/journal.pmed.0040092)
Supplement: Supplementary file 2 [file pmed.0040092.sd002.doc]

**Malaria in pregnancy: what can social sciences contribute?**

Joan Muela Ribera^1,2^, Susanna Hausmann-Muela^2^, Umberto D’Alessandro^3^, and Koen Peeters Grietens^1,2^

*^1^Universitat Autònoma de Barcelona, Departament d'Antropología Social, Bellaterra (Barcelona), Spain*

*^2^Partners for Applied Social Sciences (PASS), Belgium*

*^3^Department Parasitology, Institute Tropical Medicine, Antwerp, Belgium*

**Introduction**

Social science literature on malaria and its control is abundant. However, nearly all the publications focus on children under the age of five. Even in gender literature, women are depicted as 'mothers and caretakers of children' rather than as women suffering from malaria. The specific topic of malaria in pregnancy has received little attention in social science literature, with only some twenty articles explicitly integrating social science aspects [1-3].

Currently, the recommended intervention strategies for preventing malaria during pregnancy are Intermittent Preventive Treatment (IPT) with Sulfadoxine-Pyrimethamine (SP) and Insecticide-Treated Bed Nets (ITNs) [4]. However, in many African countries, the coverage of such interventions varies from modest to extremely low [5-6]. Although reports repeatedly mention the need to focus on behavioural aspects to better reach pregnant women, little has been done to actually promote such studies. Furthermore, though the intervention studies mention 'vulnerable groups', 'utilisation of health care services', 'delay' or 'beliefs' as important factors for effective prevention and treatment, all wordings which should immediately call social scientists onto stage, behavioural and other social science research going beyond simplistic Knowledge, Attitudes and Practices (KAP) studies are largely absent. Fortunately, the interest in social science studies on malaria in pregnancy is slowly awakening.

Social scientists should therefore occupy a more prominent role in the field of malaria in pregnancy. Building on already existing knowledge from social science work on malaria, we propose two models for studying social science aspects of malaria in pregnancy.

**What have social sciences contributed so far?**

***A. Treatment:***

Recognition of malaria and anaemia in pregnancy

With regard to children, recognition of malaria signs and symptoms has been amply studied [1, 3, 4, 7]. In general, mothers are well aware of the common malaria symptoms in their children, such as fever, vomiting and joint pain. However, not all malaria symptoms are readily attributed to the disease. For instance, convulsions are described in many studies as a distinct illness entity or 'folk illness', with its own distinct symptoms, cause and treatment [8-9]. Qualitative and quantitative data from Tanzania showed that even though a 'folk illness term' was common, the link with malaria was made by the majority of respondents [10]. A survey in Tanzania confirms that people are well aware of the link between convulsions and malaria [11].

However, the relation made by the community between malaria and anaemia was found to be low [8, 12]. Informants generally related malaria to anaemia, but the condition was not considered severe. Anaemia was associated with a childhood illness locally termed *bandama* (literally: spleen), which was loosely related to malaria, and treated traditionally [13].

While childhood malaria is extensively treated in the social science literature, almost nothing is known about community recognition of malaria and anaemia in pregnancy. One study from Uganda reports that malaria in pregnancy is not recognised as a problem [14].

***B. Prevention*:**

Chemoprophylaxis and Intermittent Preventive Treatment (IPT)

a. *Utilisation of antenatal clinics* (ANC)

ANC are the crucial point of contact for malaria prevention during pregnancy and most African countries report a high level of utilisation [15]. However, the variation between and most probably within countries is markedly high. Notably, there is a tendency for women to only attend ANC late during pregnancy, primarily in the third trimester, as has been observed in Uganda [16], Kenya [17], and Nigeria [18]. This compromises the delivery of the recommended 2-3 doses of SP given as IPT, reducing the preventive effects of this intervention [15]. Moreover, multiple ANC visits do not guarantee the multiple intake of SP. A study from Malawi reported that although most pregnant women attended antenatal services at least twice, only 36.8% received the recommended 2 doses of SP/IPT [19]

Little is known about the factors influencing access to and utilisation of ANC. Poor utilisation has been associated with education, geographic distance, lack of transport, perceived inadequacy of services, lack of privacy, and (perceived) direct and indirect high costs [16, 20-25]. Infrequent use of ANC and late first visits have also been linked to low socio-economic status, high parity, and unplanned or mistimed pregnancies [21, 26].

Most literature on ANC utilisation exists outside the malaria field. Nonetheless, such literature shows that ANC utilisation is likely to be influenced by other variables such as knowledge and perception of pregnancy-related risk factors and risk prevention. In general, recognition of risk factors in pregnancy is low [27-29] and the popular interpretation of danger signs differs from biomedical concepts [30]. Moreover, cultural concepts and notions of pregnancy and birth are likely to influence ANC utilisation. For instance, medical supervision of pregnancy is not necessarily desired by all women. In Botswana, "traditionally, pregnant women are not supposed to preoccupy themselves too concretely with their womb" [31]. Similarly, in Morocco, naturalisation of pregnancy and childbirth is common [29, 32], with the implication that monitoring and medical intervention are not seen as a priority. In Uganda, pregnancy is perceived as a natural process which does not require medical control. Not manifesting 'normal' pregnancy-related problems shows the 'braveness of the woman' [33]. This should be taken into account in societies where medicalisation of pregnancy is recent.

The traditional use of herbal medicines for pregnancy and childbirth is documented all over the world [34]. This preference may not be due to limited availability of biomedical care; indeed in South Africa women in urban areas showed the greatest interest in traditional antenatal care [34]. Furthermore, in many countries, traditional birth attendants (TBAs) play a predominant role in antenatal care and delivery [35].

Nonetheless, these socio-cultural factors which clearly influence ANC utilisation are usually not considered in malaria prevention studies. Besides reporting frequencies of a few parameters such as number of ANC visits according to different gestational ages, little attention is paid to pregnant women who never attend ANC and how to reach them. An example is found in adolescent pregnant women, who are less likely to attend antenatal care, and hence rarely reached by IPT or chemoprophylaxis [14, 36].

b. *Acceptance of chemoprophylaxis and IPT*

The most significant findings from social science studies on malaria prevention in pregnancy have been the various factors influencing poor compliance and non-acceptance of chemoprophylaxis. With regard to chloroquine (CQ), poor compliance has been related to fear of side effects (mainly itching) or perceived inefficacy of treatment [37-39]. In Malawi pregnant women were afraid of CQ, as of any other bitter drug, which they associated with damage to the foetus [40]. SP seems to be better accepted, and compliance is less problematic due to the single dose administration under supervision of health personnel. However, fears of the harmful effects of SP to woman and foetus have been reported from Uganda [14] and Tanzania [41]. Little emphasis has been placed on exploring women's understanding of perceived benefits of IPT-SP.

Adolescent pregnancies

Adolescent mothers have been identified as a particularly vulnerable group. A study in Maputo, Mozambique, found that hospitalised adolescents had a 30% higher maternal mortality rate when compared to non-adolescents [42]. This study showed that the leading cause of maternal death in adolescents was malaria, responsible for 27% of the cases, compared to only 12% in non-adolescents. In Nigeria, pregnant unmarried girls were less likely to receive antenatal care, to use health care centres for malaria treatment and to adopt appropriate measures for malaria prevention [23]. Similarly, studies from Kenya [21] and Uganda [14] report that teenagers and unmarried mothers were among those who used ANC least frequently. In a socially marketed ITN trial in Tanzania, young pregnant women, primigravidae, and unmarried pregnant women were among those with the lowest use of ITN [43].

**The malaria in pregnancy models: factors influencing malaria in pregnancy**

We propose two models for studying the social science aspects of malaria in pregnancy: the ‘Malaria in Pregnancy Treatment Model’ and the ‘Malaria in Pregnancy Prevention Model’. These are a conglomerate of different psychosocial and socio-behavioural models, based on our own field research experience and on literature review. The underlying, original models were developed for a variety of research questions, most deriving from western society. We believe that the adapted models are able to comprehensively and holistically elicit the most relevant factors involved in malaria and pregnancy.

For reasons of comprehensiveness, we have limited our models to (A) treatment and (B) prevention with IPT through ANC. The two should be considered basic, potentially generic models, adaptable to variations beyond malaria and pregnancy. The 'treatment model' can be amended for other 'single-level' interventions, where direct relations exist between the different factors, e.g. IPT through community-based channels, chemoprophylaxis, or other drug interventions. The 'prevention model' lays the basis for addressing 'multiple-level' interventions, where different interventions relate to and might influence each other, e.g. new interventions added to the Expanded Program of Immunization (EPI).

The variety in ITN distribution approaches (social marketing, voucher systems, community-based programs, shopkeepers etc.) makes analysing malaria prevention with ITN more complex. We therefore exclude ITN from the models, although many of the factors would apply to ITN too.

The added value of the proposed models when compared to other sociocultural work about malaria and pregnancy is that a set of factors rather a single one are considered. They are therefore based on a selection of elements, all intricately interwoven, from different psychosocial and behavioural models applied for general malaria prevention and treatment-seeking behaviour, [13] and further developed for malaria in pregnancy (Figures 1 & 2). The models comprise socio-demographic and sociocultural variables, including recognition and perception factors, the ‘A factors’, i.e. availability, accessibility, affordability, and the time lost due to prevention and treatment-seeking. While availability and accessibility are primarily health provider factors, not under direct influence of the users (pregnant women), all the other factors are user-related.

***A. The Treatment Model***

*1. Sociocultural and demographic variables and social context*

The sociocultural and demographic variables help to distinguish different groups, according to age/age group, number of pregnancies, socio-economic status, marital status, religion and magico-religious beliefs, ethnicity, and can include other factors which may differentiate groups of people according to relevant criteria. The list is not complete, but rather gives the classical variables that serve as a basis for designating specific social categories that can be compared and possibly individually targeted. Depending on the specific setting, other sociocultural and demographic variables might be relevant.

*2. Recognition of malaria and anaemia during pregnancy*

While community knowledge about the malaria-mosquito link can be considered fundamental in determining the use of ITNs, prompt and effective treatment depends on illness recognition. Yet, malaria-related symptoms can be easily confused with pregnancy-related symptoms. No publication was found that explores the way pregnant women distinguish malaria signs from general malaise and other common symptoms (e.g. nausea, vomiting, weakness, etc.) during pregnancy. Furthermore, there is the need to know whether pregnant women’s susceptibility to anaemia is of common knowledge; and if people make the link between anaemia during pregnancy and malaria.

*3. Perceived severity*

Perceived severity is a key factor in the *Health Belief Model* [44] –the most widely used model in public health-, in socio-behavioural models [45-46] and in anthropological decision-making models [47]. Studies have shown that malaria is often not perceived as severe, but rather as a mild, self-limiting illness which does not require immediate treatment [8, 12]. In this way, studies should focus on (1) the perceived severity of malaria for the mother, with emphasis on knowledge regarding anaemia and maternal mortality risk; (2) for the foetus, and the recognition of the risk for abortion; and, (3) the perceived severity of the illness for the newborn, including the association of low birth weight and increased vulnerability to other illnesses.

*4. Perceived susceptibility*

Perceived susceptibility is another key factor taken from the *Health Belief Model*. With regard to malaria, perceived susceptibility is related to two factors: (1) the perceived propensity to develop clinical malaria due to idiosyncratic features of the person (pregnant women, children, weak persons); and (2) the perceived level of exposure. For instance, where malaria is associated with mosquitoes and rainfall [48- 49], perceived susceptibility of contracting malaria seems to be strongly related with mosquito density and the rainy season [50]. These factors necessarily lead to two research questions regarding perceived susceptibility to malaria during pregnancy: (1) whether pregnant women are considered particularly susceptible to malaria and (2) when are they perceived to be more susceptible (i.e. related to seasonality or to risk activities).

*5. Perceived benefits*

Perceived benefits of treatment (or preventive measures) are another important factor of the *Health Belief Model*. Perceived benefits need to be studied in relation to (1) the perceived efficacy of a product or an intervention (determined not only by the empirical experience, but also by the persuasiveness of the message); and (2) the perceived costs/benefits, understood not only in economically and health related terms, but also socially and psychologically. In this sense, factors such as the evaluation of the distance to treatment facilities, the waiting time or the behaviour of health staff can play an important role.

It is also important to consider the perceived *complementary* benefits of a specific treatment. ITNs are a classical example because they are perceived as additionally beneficial for avoiding the nuisance of mosquito bites rather than for preventing malaria [48]. Hence, comfort rather than health constitutes the perceived benefit.

Similar to perceived severity, the perceived benefits can be identified (1) for the mother; (2) for the foetus; and, (3) for the newborn. It is important to know whether socially the mother or the foetus is prioritized since the benefits for one might imply risks for the other.

Perceived benefits should always be studied taking into consideration their counterpart, the perceived risks.

*6. Perceived risks*

Perceived risks of treatment are among the central factors for understanding treatment acceptance and use. Just as with perceived severity and perceived benefits, perceived risks refer to mother, foetus and newborn. Perceived risks are related to (1) perceived iatrogenic effects of treatment; (2) perceived side-effects of antimalarials; and (3) perceived risks of under- and overdosage of antimalarials. An example of perceived risks is the fear of adverse drug reactions if the pregnant woman is possessed by a spirit that rejects western pharmaceuticals.

Additionally, the risk from the biomedical perspective might not be considered a real threat by the affected individual or the perceived risk may be offset by perceived complementary benefits. For example, in Burkina Faso preliminary qualitative information suggests that women prefer low birth weight for their babies since they are considered easier to deliver and ‘small babies’ have the perceived benefit of reducing the risk of episiotomy. Women state they prefer ‘*the baby to grow after giving birth instead of before*’ (Peeters Grietens, personal communication).

*7. Perceived control and decision-making*

Perceived control and decision-making should not be understood as a factor but rather as a space where different factors come into play. A tension exists, and must be taken into consideration, between (1) the perceived control over action, determined by perceived and real access to the necessary resources for successful action (information, assets, abilities, social networks, opportunities etc.), and the value attributed to these resources [51] and (2) the perceived obstacles and structural limitations (social, institutional, economic) to the planned action. In order to elaborate on this, we must focus on who decides – the *Therapy Management Group* [52]. For instance, when women as the main caretakers recognize illness but decision-making and control over resources (i.e. money for covering transport costs) are in the hands of their husbands, possibly having other priorities, perceived necessity and action do not correspond, presenting a similar situation to that described for childhood malaria [53].

It is important to study how decisions for malaria treatment-seeking in pregnant women are made, to understand the intra-household hierarchies and the criteria implied in decision-making. In this way, participation may be strongly influenced by others than those directly targeted. It is also important to take into account the social pressure for complying with the treatment (or preventive measures), determined by gender ideologies which can result in stigmatization ('the bad mother'), and socio-moral perceptions about ‘appropriateness’ and ‘responsibility’ [54-55].

*8. Availability, 9. accessibility and 10. time loss*

Availability, accessibility and time loss are important factors accounting for therapeutic delay. Availability implies, among other things, that the health facilities are equipped with drugs and are recognised as competent for diagnosis and treatment. Concerning access to treatment, it should be noted that rural women may need to work and live on fields situated far away from the health centres and, during the rainy seasons, roads may be inaccessible, hindering access to health centres. The perception of time lost travelling to and from the health centre or waiting at the health centre clearly influences treatment seeking in relation to the labour situation of the women, with regard to child care and intra-domestic labour substitution. Perception of time lost pertains to perceived and real loss of productivity during treatment-seeking and, at the same time, is linked to the perceived impact and severity of the illness.

*11. Direct and indirect costs*

The few studies that contemplate costs only consider the medical costs involved in treatment [e.g. 56]. However, other non-medical direct and indirect costs are essential to understand the total costs of a malaria episode. Such non-medical direct costs consist of transportation costs, costs for the accompanying relatives, food for the patient and visitors, payment for labour substitution, etc. Indirect costs refer to productivity loss of the patient and caretaker(s), encompassing those due to time lost travelling to a health centre and work lost while hospitalised, both for patients and their caretakers [57-58].

The general costs for pregnant women are especially relevant because (1) women are at the same time caretaker, worker and patient; and, (2) low-birth weight infants are more likely to contract illnesses, meaning a considerable increase in health care expenditures for the household.

For illnesses in general it is important not only to take into account the costs, but also the strategies that women (and/or their families) can activate to cope with illness and treatment-seeking costs [58]. Strategies to cope with elevated costs can imply no action [59] to minimize the immediate economic impact of illness; the mobilisation of social networks; selling *tangible assets* (animals, domestic or agricultural tools, houses, fields, stored food, cash money, etc.); and, using *intangible assets,* such as claims for help or support to other households or relatives, to patrons, elders or leaders, to the government or the international community [60], and involve *access* or real opportunity to utilize the resources, to obtain information or help [61].

***B. Prevention with IPT***

Prevention with IPT represents an intervention that encompasses two levels, the administration of IPT and the structure of antenatal care (ANC) (Figure 2). For the IPT part, the above mentioned factors 1-7 practically remain unaltered and are not repeated here. There are only a few alterations with regard to antimalarials used as a preventive rather than treatment measure.

Factors 12-15 in Figure 2, availability, accessibility, time loss and cost factors are similar to those mentioned in the treatment model (Figure 1), with the difference that they directly relate to ANC rather than to malaria treatment and perceived adequacy of curative care. It is worth emphasising that even when ANC is free of charge, direct non-medical and indirect costs, especially those for transport, are still considerable and might hinder access.

In the following section, we describe the factors relevant at the ANC level, the factors 8-12 in Figure 2.

8. Social values, perception and attitudes towards pregnancy and pregnancy-related risks

ANC-facilities are *public spaces*, where women unavoidably reveal their pregnancy to other community members. Fear of gossip and humiliation due to socially unacceptable and shameful pregnancies, e.g. adolescent or out of wedlock pregnancies, can be a reason for delaying prenatal care, or even deterring pregnant women from attending ANC (Peeters Grietens, unpublished report). Another important reason for avoiding public exposure of their pregnancy during the first months is the fear of sorcery-related harm to mother or foetus [62].

A further key factor is the perception of pregnancy and pregnancy-related risks. These perceptions are based on (1) cultural models of the physiology of pregnancy and foetal growth, including the symptoms of 'normal' pregnancy and symptoms of alert; (2) social values concerning 'appropriate' behaviour, e.g. if it is socially acceptable to publicly show suffering or fatigue. Perceptions of risks together with perceived benefits constitute the *perceived need* to attend ANC.

9. Perceived benefits of antenatal care

It is important to know women's perception of the different services offered at ANC – control of foetal development; control of infectious diseases (AIDS and other STDs etc.); preventive measures such as administration of iodine, iron and folic acid complements; tetanus immunization; breastfeeding counselling and birth preparation – since some of these services might be valued more than others. In this sense, while some might encourage, others might discourage ANC attendances.

Perceived benefits of ANC should be studied taking into consideration perceived obstacles for attendance. Obstacles, e.g. work load, lack of transport, limited amount of money, may play a stronger role for routine visits than for visits under circumstances of perceived risk.

10. Acceptability of and satisfaction with antenatal services

Acceptability, particularly satisfaction with ANC, is usually related to (1) the perceived behaviour of health personnel; and (2) the perceived quality of services. Common complaints on health personnel misbehaviour include claims of medical neglect, patient humiliation and rudeness at the hands of medical staff [63]. Low acceptability can also be related to ethnic or class differences between health staff and patients and can result in misunderstandings and mutual mistrust. Communication barriers due to different vernacular languages or to women's fear – particularly among Muslims – of being attended by male nurses can further discourage ANC attendance [26].

11. Social support for attending ANC

With regard to access to ANC, social network support has two important dimensions: (1) social support: when giving advice to the pregnant woman; participating in pregnancy rituals where they exist; accompanying the woman to antenatal services etc.; and, (2) economic support: contributing to ANC costs and other direct and indirect costs. It is important to know who participates in which dimension, who decides to attend ANC and how these decisions are reached. Since pregnancy is a family matter, both dimensions must be understood in the framework of social organisation of procreation, and of gender and kinship ideologies. Lack of social support can be due to various factors, e.g. impoverished households, absent 'relevant others' or poor social pressure for the husband to pay [25].

Interference

The great difference of the prevention model when compared to the 'treatment model' is the overlapping of ANC utilisation and IPT acceptance and the probability of interaction between both sets of variables.

Logistically, the combination of control methods like the integration of IPT into ANC structures is highly advantageous. However, the effects of one on the other are often underestimated. From the health provider side, the workload and required knowledge might mean a considerable additional burden for the health personnel. From the pregnant women’s side, interferences are likely but largely unknown (in the figure depicted as 'interference'). Indications that such interference and community response exist comes from a study carried out in Malawi where community distribution of IPT through peer educators from the adolescent literacy programme led to a decrease in formal ANC attendance and hospital delivery (Bernard Brabin, personal communication). Similarly, IPT delivered through ANC clinics might encourage or discourage further attendance and influence women's perception of contracting malaria in pregnancy.

***Conclusions***

We have proposed two models which encompass the different social factors that influence health seeking behaviour for malaria in pregnant women and demonstrate how they are related to each other. Together with quantitative analysis which evaluates the relative occurrence of the different factors and ranks them according to their frequency, qualitative analysis is incorporated to contextualize the factors, put them into a dynamic relation and assess their relative weight and importance within the general social structure. We have developed these models for malaria in pregnancy because it is in this field that social sciences are particularly neglected. Research on and implementation of malaria control intervention for pregnant women have predominantly ignored community responses or, when considered, they have centred on single, isolated factors usually with the aim of designing 'culturally sensitive' Information, Education, Communication (IEC) messages. Reality, however, shows that the implementation of 'simple' tools, like IPT delivery to pregnant women, is not so simple because community reactions are not taken into account.

With this article, we hope to create awareness among researchers of such complex interactions and the need of involving social sciences even for apparently ‘straightforward' interventions. Studying these interactions may help to improve the delivery of adequate interventions and thus contribute to reaching the Abuja Malaria Summit target of at least 60% of pregnant women adequately protected against malaria infection and its consequences. These models have to be taken as a first contribution to concisely cover social science aspects. We hope that they help to inspire future works in the malaria social science literature, particularly in the under researched field of malaria and pregnancy.

***Acknowledgements***

We acknowledge the reviewers Brigit Obrist and Imelda Bate for their valuable input. This article is part of the "New approaches to improve coverage and compliance of antimalarial treatment for pregnant women in rural Africa" study financed by the European Commission in the framework of the cost-shared concerted action on malaria and anaemia control in pregnant women “PREMA-EU” (contract number IC-CT-2001-10012).

***References***

1. McCombie, S. C. (1996) Treatment seeking for malaria: a review of recent research. *Social Science and Medicine* **43**: 933-945.
2. Williams, H. A. and Jones, C. (2004) A critical review of behavioural issues related to malaria control in sub-Saharan Africa: what contributions have social scientists made? *Social Science and Medicine* **59**: 501-523.
3. Heggenhougen, H. K., Hackenthal, V. and Vivek, P. (2003) *The behavioural and social aspects of malaria and its control. An introduction and annotated bibliography*. UNDP/World Bank/WHO.
4. World Health Organization (2004) *A strategic framework for malaria prevention and control during pregnancy in the African region*. Brazzaville: WHO Regional Office for Africa AFR/MAL/04/01.
5. Holtz, TH; Kachur, S.P., Roberts, J.M., Marum, L.H., Mkandala, C., Chizani, N., Macheso, A., Parise, M.E. (2004) Use of antenatal care services and intermittent preventive treatment for malaria among pregnant women in Blantyre District, Malawi. *Trop Med Int Health*. **9**: 77-82.
6. Guyatt, H.L., Noor, A.M., Ochola, S.A., Snow, R.W. (2004) Use of intermittent presumptive treatment and insecticide treated bed nets by pregnant women in four Kenyan districts. *Trop Med Int Health*. **9**: 255-261.
7. Clarke, S. E. and Svenningsen, E. (2003) (Eds.) *People and malaria medicines: use of antimalarials in the home*. Proceedings of a Workshop held in Mbarare, Uganda, August 2001, Danish Bilharziasis Laboratory.
8. Mwenesi, H., Harpham, T., and Snow, R.W. (1995) Child malaria treatment practices among mothers in Kenya. *Social Science and Medicine* **40**: 1271-1277.
9. Makemba, A. M., Winch, P. J., Makame, V. M., Mehl, G. L., Premji, Z., Minjas, J. N., and Siff, C. J. (1996) Treatment practices for degedege, a locally recognized febrile illness, and implications for strategies to decrease mortality from severe malaria in Bagamoyo District, Tanzania. *Trop Med and Int Health* **1:** 305-313.
10. Hausmann-Muela, S. & Muela Ribera, J. (2003) Recipe knowledge : a tool for understanding some apparently irrational behaviour. *Anthropology & Medicine*, **10**: 87-105.
11. De Savigny, D., Mayombana, C., Mwageni, E., Masanja, H., Minhaj, A., Mkilindi, Y., Mbuya, C., Kasale, H. and Reid, G. (2004) Care-seeking patterns for fatal malaria in Tanzania. *Malaria Journal* **3**: 27.
12. Winch, P.J., Makemba, A.M., Kamazima, S.R., Lurie, M., Lwihula, G.K., Premji, Z., Minjas, J.N., and Shiffe, C.J. (1996) Local terminology for febrile illnesses in Bagamoyo District, Tanzania, and its impact on the design of a community based malaria control programme. *Social Science and Medicine* **42**: 1057-1067.
13. Hausmann-Muela, S., Muela Ribera, J. & Nyamongo, I. (2003) *Health-Seeking Behaviour and the Health System Response*, DCPP (Disease Control Priorities Project), DCPP Working Papers Series. http://www.fic.nih.gov/dcpp/wps.html.
14. Mbonye, A. K., Neema, S. and Magnusen, P. (2005) Preventing malaria in pregnancy: a study of perceptions and policy implications in Mukono district, Uganda. *Health Policy and Planning* **21**: 17-26.
15. World Health Organization and UNICEF (2004) Antenatal care in developing countries: promises, achievements and missed opportunities. Geneva: World Health Organization and United nations Children’s Fund.
16. Ndyomugyenyi, R., Neema, S. and Magnussen, O. (1998) The use of formal and informal services for antenatal care and malaria treatment in rural Uganda. *Health Policy and Planning* **13**: 94-102.
17. Hamel, M.J., Odhacha, A., Roberts, J.M., and Deming, M.S. (2001) *Bull World Health Organization* **79**: 1014-1023.
18. Brieger, W. R., Luchok, K.J., Eng, E., and Earp, J.A. (1994) Use of maternity services by pregnant women in a small Nigerian community. *Health Care Women International* **15**: 101-110.
19. Holth, T.H., Kachur, P., Roberts, J.M., Marum, L.O., Mkandala, C., Chizani, N., Macheso, A. and Parise, M.E. (2004) Use of antenatal care services and intermittent preventive treatment for malaria among pregnant women in Blantyre District, Malawi. *Tropical Medicine and International Health* **9(1)**: 77-82.
20. Schultz, L.J., Steketee, R.W., Chitsulo, L., Macheso, A., Nyasulu, Y., and Ettling, M. (1994) Malaria and childbearing women in Malawi: knowledge, attitudes and practices. *Tropical Medical Parasitology* **45**: 65-69.
21. Magadi, M.A., Madise, N.J., and Rodrigues, R.N. (2000) Frequency and timing of antenatal care in Kenya: explaining the variations between women of different communities. *Social Science and Medicine* **51**: 551-561.
22. Materia, E., Mehari, W., Mele, A., Rosmini, F., Stazi, M.A., Damen, H.M., Basile, G., Miuccio, G., Ferrigno, L., and Miozzo, A., (1993) A community survey on maternal and child health service utilization in rural Ethiopia. *European Journal of Epidemiology* **9**: 511-516.
23. Acharya, L.B. & Cleland, J. (2000) Maternal and child health services in rural Nepal: does access or quality matter more? *Health Policy and Planning* **15**: 223-229.
24. Okonofua, F. E., Feyisetan, B. J., Davies-Adetugbo, A. and Sanusi, Y. O. (1992) Influence of socio-economic factors on the treatment and prevention of malaria in pregnant and non-pregnant adolescent girls in Nigeria. *Journal of Tropical Medicine and Hygiene* **95**: 309-315.
25. Telfer, M.L., Rowley, J.T. and Walraven, G.E.L. (2002) Experiences of mothers with antenatal, delivery and postpartum care in rural Gambia. *African Journal of Reproductive Health*, **6(1)**: 74-83.
26. Zanconato, G., Msolomba, R., Guarenti, L. and Franchi, M. (2006) Antenatal care in developing countries: The need for a tailored model. *Semminars in Fetal and Neonatal Medicine*, **11(1)**: 15-20.
27. Mlay, R., Massawe, S., Lindmark, G. and Nystrom, L. (1994) Recognition of risk factors in pregnancy among women attending antenatal clinic at Mbagala, Dar-es-Salaam. *East African Medical Journal* **71**: 562-566.
28. Oosterbaan, M.M. and da Costa, M.V. (1995) Guinea-Bissau: what women know about the risks – an anthropological study. *World Health Statistics Quarterly* **48**: 39-43.
29. Dialmy, A. (1999) *La gestion socioculturelle de la complication obstétricale dans les régions Fès – Boulement et Taza – Al Hoceima – Taounate (Maroc).* Ministère de la Santé, Direction de la Population, Maroc.
30. Kowalewski, M, Jahn, A., and Kimatta, S.S. (2000) Who do at-risk mothers fail to reach referral level? Barriers beyond distance and cost. *African Journal of Reproductive Health* **4**: 100-109.
31. Tautz, S., Jahn, A., Molokomme, I., and Görgen, R. (2000) Between fear and relief: how rural pregnant women experience foetal ultrasound in a Botswana district hospital. *Social Science and Medicine* **50**: 698-701.
32. Makhlouf Obermeyer, M. (2000) Pluralism and pragmatism: knowledge and practice of birth in Morocco. *Medical Anthropology Quarterly* **14**: 180-201.
33. Kyomuhendo, G.B. (2003) Low use of rural maternity services in Uganda: Impact of women’s status, traditional beliefs and limited resources. *Reproductive Health Matters*, **11(21)**: 16-26.
34. Varga, C. A. and Veale, J. H. (1997) *Isihlambezo*: Utilization patterns and potential health effects of pregnancy-related traditional herbal medicine. *Social Science and Medicine* **44**: 911-924.
35. Helman, C. (2001) *Culture, health and illness*. London: Arnold Publishers.
36. D’Alessandro, U. (1999) A rational approach to malaria control in sub-Saharan Africa: The need for a link between scientific research and public health interventions. *Annals of Tropical Medicine & Parasitology* **93** (Suppl. 1): S75-S77.
37. Kaseje, D.C., Sempebwa, E.K., and Spencer, H.C. (1987) Malaria chemoprophylaxis to pregnant women provide by community health workers in Saradidi, Kenya. I. Reasons for non-acceptance. *Annals of Tropical Medical Parasitology* **81** (Suppl.1): 77-82.
38. Mnyka, K. S., Kabalimu, T. K. and Lugoe, W. L. (1995) Perception and utilization of malaria prophilaxis among pregnant women in Dar es Salaam, Tanzania. *East Africa Medical Journal* **72**: 431-435.
39. Parise, M. E., Lewis, L. S., Ayisi, J. G., Nahlen, B. L., Slutsker, L., Muga, R., Sharif, S. K., Hill, J. and Steketee, R. W. (2003) A rapid assessment approach for public health decision-making related to the prevention of malaria during pregnancy. *Bull World Health Organization* **81**: 316-323.
40. Helitzer-Allen, D.L. and Kendall, C. (1992) Explaining differences between qualitative and quantitative data: a study of chemoprophylaxis during pregnancy. *Health Education Quarterly* **19**: 41-54.
41. Mubyazi, G., Bloch, P, Kamugisha, M., Kitua, A. and Ijumba, J. (2005) Intermittent preventive treatment of malaria during pregnancy: A qualitative study of knowledge, attitudes and practices of district health managers, antenatal care staff and pregnant women in Korogwe District, North-East Tanzania. *Malaria Journal* 4:31.
42. Granja, A. C., Machungo, F., Gomes, A. and Bergström, S. (2001) Adolescent maternal mortality in Mozambique. *Journal of Adolescent Health* **28**: 303-306.
43. Marchant, T.J., Armstrong Schellenberg, J, Edgar, T., Nathan, R., Abdulla, S., Mukasa, O., Mbonda, H., and Lengeler, C. (2002) Socially-marketed insecticide-treated bednets improve malaria and anaemia in pregnancy in southern Tanzania. *Trop Med & Int Health* **7:** 149-158.
44. Sheeran, P. & Abraham, C. (1995) The Health Belief Model, in *Predicting Health Behaviour* (Conner, M. & Norman, P. eds.). Buckingham: Open University Press.
45. Andersen, R., Kravits, J., and Anderson, O.W. (eds.) (1975) *Equity in health services: empirical analyses in social policy.* Cambridge Massachussetts: Ballinger.
46. Kroeger, A. (1983) Anthropological and socio-medical health care research in developing countries. *Social Science & Medicine,* **17**:147-161.
47. Weller, S.C., Ruebush II, T.R. & Klein, R.E. (1997) Predicting treatment-seeking behaviour in Guatemala: A comparison of the Health Services research and Decision-Theoretic approaches. *Medical Anthropology Quarterly,* **11**: 224-245.
48. Minja, H. (2001) *Introducing insecticide treated mosquito nets in the Kilombero Valley (Tanzania): social and cultural dimensions*. PhD thesis, Swiss Tropical Institute, University of Basel.
49. Winch, P.J., Makemba, A.M., Kamazima, S.R., Lurie, M., Lwihula, G.K., Premji, Z., Minjas, J.N., and Shiff, C.J. (1994) Seasonal variation in the perceived risk of malaria: implications for the promotion of insecticide-treated nets. *Social Science and Medicine* **39**: 63-75.
50. Sauerborn, R., Nougtara, A., Hien, M. and Diesfeld, H. J. (1996) Seasonal variations of household costs of illness in Burkina Faso. *Social Science and Medicine* **43**: 281-290.
51. Conner, M. & Sparks, P. (1995) The Theory of Planned Behaviour and Health Behaviours, in *Predicting Health Behaviour* (Conner, M. & Norman, P. eds.). Buckingham: Open University Press.
52. Janzen, J.M. (1978) *The Quest for Therapy in Lower Zaire*. Berkeley: University of California Press.
53. Mwenesi, H. (1993) *Mother’s definition and treatment of childhood malaria on the Kenyan coast*. PhD thesis. London School of Hygiene and Tropical Medicine.
54. De Martin, S., Seidlein, L, Deen, J.L., Pinder, M., Walraven, G., and Greenwood, B. (2001) Community perceptions of a mass administration of an antimalarial drug combination in The Gambia. *Trop Med & Int Health* **6**: 442-448.
55. Ager, A. (1992) Perception of risk for malaria and schistosomiasis in rural Malawi. *Tropical Medical Parasitology* **43**: 234-238.
56. Manzi, F., Armstrong Schellenberg, J., Adam, T., Mshinda, H., Victoria, C.G. and Bryce, J. (2005) Out-of-pocket payments for under-five health care in rural southern Tanzania. *Health Policy and Planning*, **20**: 85-93.
57. Russell, S. (2004) The economic burden of illness for households in developing countries: A review of studies focusing on malaria, tuberculosis and Immunodeficiency Virus/Acquired Immunodeficiency Syndrome. *Am J Trop Med and Hyg.* **7** (Suppl. 2): 147-155.
58. McIntyre, D., Thiede, M., Dahlgren, G. and Whitehead, M. (2006) What are the economic consequences for households of illness and of paying for health care in low- and middle-income country contexts? *Social Science and Medicine,* **62**(4):858-865*.*
59. Ryan, G.W. (1998) What do sequential behavioural patterns suggest about the medical decision-making process?: Modeling home case management of acute illnesses in a rural Cameroonian village. *Social Science & Medicine,* **46**:209-225.
60. Swift, J. (1989) Why are rural people vulnerable to famine? in Vulnerability: How the Poor Cope (Chambers, R. ed.). *IDS Bulletin,* **20**.
61. Chambers, R. (1995) *Poverty and livelihoods: Whose reality counts?* Brighton: Institute of Development Studies, University of Sussex, Discussion paper 347.
62. Mainbolwa, M., Yamba, B., Divan, V. and Ransjö-Arvidson, A.B. (2003) Cultural childbirth practices and beliefs in Zambia. *Issues and Innovations in Nursing Practices*, **43(3)**: 263-274.
63. Pires Lucas d’Oliveira, A.F., Grilo Diniz, S. and Blima Schraiber, L.B. (2002) Violence against women in health-care institutions: An emerging problem. *The Lancet*, 359(9318): 1681-1685.

Figure 1. The malaria in pregnancy treatment model: relevant factors for treatment-seeking behaviour for malaria in pregnant women

2. Recognition of malaria and anaemia during pregnancy

3. Perceived severity of malaria and anaemia in pregnancy

4. Perceived susceptibility to malaria in pregnancy

5 Perceived benefits of antimalarials in pregnancy

6. Perceived risks of antimalarials in pregnancy

8. Availability

9. Accessibility

10. Time loss

11.Costs

1. Sociocultural and socio- demographic variables

&

Social context

7. Perceived control and actual capacity for action (the social context of decision making and action)

**TREATMENT**

Figure 2. The malaria in pregnancy prevention model: relevant factors for the utilization of ANC and acceptance of IPT

1. Sociocultural and socio-demographic variables

&

Social context

2. Recognition of malaria and anaemia during pregnancy

3. Perceived severity of malaria in pregnancy for woman and foetus

4. Perceived susceptibility of malaria in pregnancy

5. Perceived benefits of antimalarials in pregnancy as a preventive measure

12.Availability

15. Costs

14. Time loss

13.Accessibility

9. Perceived benefits of antenatal care

10. Acceptability and satisfaction with antenatal services

11. Social support for attending antenatal care

6. Perceived risks of antimalarials in pregnancy as a preventive measure

**Interference**

7. Perceived control and actual capacity for action

(the social context of decision-making and action)

8. Social values and perceptions towards pregnancy and pregnancy-related risks

Availability and correct administration

Antenatal care

Intermitent Preventive Treatment
